# Exogenous Application of Calcium Ameliorates Salinity Stress Tolerance of Tomato (*Solanum lycopersicum* L.) and Enhances Fruit Quality

**DOI:** 10.3390/antiox12030558

**Published:** 2023-02-23

**Authors:** Md. Moshiul Islam, Khurshida Jahan, Arpita Sen, Tahmina Akter Urmi, M. Moynul Haque, Hayssam M. Ali, Manzer H. Siddiqui, Yoshiyuki Murata

**Affiliations:** 1 Department of Agronomy, Faculty of Agriculture, Bangabandhu Sheikh Mujibur Rahman Agricultural University, Gazipur 1706, Bangladesh; 2 Bangladesh Institute of Nuclear Agriculture (BINA), Mymensingh 2202, Bangladesh; 3 Department of Soil Science, Faculty of Agriculture, Bangladesh Agricultural University, Mymensingh 2202, Bangladesh; 4 Department of Botany and Microbiology, College of Science, King Saud University, Riyadh 11451, Saudi Arabia; 5 Graduate School of Environmental and Life Science, Okayama University, 1-1-1 Tsushima-Naka, Okayama 700-8530, Japan

**Keywords:** NaCl stress, calcium, reactive oxygen species, antioxidant enzyme activity, fruit quality

## Abstract

Tomato is affected by various biotic and abiotic stresses, especially salinity, which drastically hinders the growth and yield of tomato. Calcium (Ca) is a vital macronutrient which plays physiological and biochemical roles in plants. Hence, we studied the protective roles of Ca against salinity stress in tomato. There were eight treatments comprising control (nutrient solution), 5 mM Ca, 10 mM Ca, 15 mM Ca, 12 dS m^−1^ NaCl, 12 dS m^−1^ NaCl + 5 mM Ca, 12 dS m^−1^ NaCl + 10 mM Ca and 12 dS m^−1^ NaCl + 15 mM Ca, and two tomato varieties: BARI tomato-2 and Binatomato-5. Salinity significantly decreased the plant-growth and yield attributes, relative water content (RWC), photosynthetic pigments (SPAD value) and the uptake of K, Ca and Mg in leaves and roots. Salinity-induced oxidative stress was present in the form of increased Na^+^ ion concentration, hydrogen peroxide (H_2_O_2_) content and lipid peroxidation (MDA). Ca application reduced oxidative stress through the boosting of antioxidant enzymatic activity. Exogenous Ca application enhanced proline and glycine betaine content and reduced Na^+^ uptake, which resulted in the inhibition of ionic toxicity and osmotic stress, respectively. Hence, Ca application significantly increased the growth and yield attributes, RWC, SPAD value, and uptake of K, Ca and Mg. Calcium application also had a significant effect on the fruit quality of tomato and the highest total soluble solid, total sugar, reducing sugar, β-carotene, vitamin C and juice pH were found for the combined application of NaCl and Ca. Therefore, application of Ca reversed the salt-induced changes through increasing osmoprotectants, activation of antioxidants enzymes, and by optimizing mineral nutrient status.

## 1. Introduction

Global agriculture feeds over seven billion people and this number is expected to increase by a further 50% by the year 2050 [1]. To meet the additional food demand for this growing population, crop production should increase between 70% and 100% by 2050 [2]. On the other hand, globally, the availability of agricultural land is shrinking gradually due to a rapid rise in industrialization, urbanization, and biotic and abiotic stresses [3]. Among the abiotic stresses, salinity is increasing around the globe due to the intrusion of salt water in crop fields as a result of sea-level rises in coastal areas, extensive irrigation practices, and large-scale soil erosion [4]. It is estimated that approximately 7% of the world’s total land area and 20% (more than 830 million ha) of the gross cultivable area are affected by different levels of salinity [5]. Thus, salinity is a major abiotic stress which restricts crop yield and sustainable agricultural production.

Salinity causes ionic, oxidative and osmotic stress which retards plant growth and development. Salinity restricts water absorption by the plant roots, which leads to osmotic stress [6,7]. Due to the restriction of nutrient uptake under salt stress, plants suffer from nutrients deficiency. In addition, due to enhancing Na^+^ influx and K^+^ efflux which leads to an elevated Na^+^/K^+^ ratio in plant cells, plants suffers from ionic stress [8,9]. High salt concentrations, particularly higher Na^+^ concentrations in the transpiration stream of plants, can be injurious to cells, resulting in the inhibition of several physiological and biochemical routes such as nutrient uptake (such as potassium, calcium and magnesium) and CO_2_ assimilation [10]. In addition, the accumulation of toxic ions negatively regulates plant–water relations and degrades photosynthetic pigments which trigger lower transpiration rates, photosynthesis, growth and biomass production [11].

Salinity is also responsible for oxidative stress, which generates reactive oxygen species (ROS), which oxidize biomolecules such as nucleic acids, proteins, lipid bilayer membranes, and enzyme inhibitors [12]. Immediately after stress exposure, plants respond to the stress stimuli and transmit signals for physiological and biochemical changes for survival and growth [13]. Thus, plants produce secondary metabolites and regulate plant physiological processes to manage salt-induced stress [14]. Moreover, plants produce different compatible osmoprotectants such as proline, glycine betaine, and sucrose, which helps to maintain water relations, and stabilize enzymes, protein complexes and membranes under saline condition [15,16]. Furthermore, plant defense against increased oxidative stress is coupled with the maintenance of a cellular redox equilibrium, which is mostly conferred by some enzymatic and non-enzymatic antioxidants [17,18].

Tomato (*Solanum lycopersicum* L.) is an important crop worldwide and one of the most consumed vegetables in the world. Tomato is considered one of the most important “protective foods” because of its special nutritive value. Tomato is rich in carotenoids such as β-carotene, lycopene and lutein. β-carotene is a precursor of vitamin A. Lycopene reduces the risk of cancer, cardiovascular disease and macular degeneration. Lutein plays a vital role in the protection of vision, and in preventing age-related maculopathy [19,20]. Furthermore, tomato is also a source of vitamins and phenolic compounds. All of these compounds contribute to tomato’s antioxidant properties and beneficial health effects [21]. The above-mentioned compounds can be affected by environmental factors [20]. The effect of salinity on tomato fruit has been well-documented, indicating a decrease in yield and changes in fruit quality [20]. Moreover, using controlled abiotic stress may be an interesting approach to improving the nutraceutical value of tomato fruits [22].

In most cases, genetic self-defense capacity is not enough to fully secure plants from oxidative damage. Plant scientists are looking for alternatives to promote plants’ ability to survive under diverse environmental stresses. Recently, exogenous use of small biological molecules such as phytohormones and signaling molecules have become popular. Calcium (Ca) is a prime macronutrient in plants as well as a universal secondary messenger in plant signaling. Activation of influx channels both in the plasma membrane and tonoplast increases Ca content in the cytosol and plays a vital role in the enhancement of abiotic stress tolerance in plants [23,24]. By making bonds with the phospholipid bilayer, Ca regulates the structure, signaling and function of membranes and, hence, stabilizes and promotes the structural integrity of membrane organelles in plants under stress environments [25]. In addition, Otie et al. [5] and Tan et al. [26] reported that Ca restricts the entry of Na^+^ into plant cells under sodium stress. Therefore, exogenous application of Ca is an important method to overcome the salt stress. Several previous studies also reported that exogenous calcium mitigates salt stress in tomato by osmotic adjustment, increasing antioxidant enzyme activities and the accumulation of sodium, potassium, proline and by enhancing root and shoot formation [27,28,29]. However, many aspects of exogenous Ca-mediated salt tolerance in tomato remain elusive. With this background, the present research work was, therefore, performed to analyze the potential roles and possible mechanisms of Ca-mediated salt-stress tolerance in tomato. Different agronomic, physiological, biochemical and fruit-quality determinants of salinity tolerance were assessed to examine the salt-stress relief mechanism by Ca.

## 2. Materials and Methods

### 2.1. Plant Material and Treatment

Two tomato (*Solanum lycopersicum* L.) varieties—BARI tomato-2 (high yielding, salt-tolerant) and Binatomato-5 (high yielding, salt-susceptible)—were collected from Plant Breeding Division, Bangladesh Institute of Nuclear Agiculture (BINA), Mymensingh, Bangladesh and Bangladesh Agriculture Research Institute (BARI), Gazipur, Bangladesh [30]. Experiment was conducted at the vinyl house with temperature of 25 °C, 60% relative humidity and 16 h light period with 80 μmol m^−2^ s^−1^ photon flux density and 8 h dark period. The seeds were surface-sterilized with 2.5% sodium hypochlorite for 15 min and washed thoroughly with distilled water several times. Tomato seeds were germinated on filter paper in Petri plates (9 cm diameter) in a germination chamber. After germination, seedlings were transferred to 20 L plastic pots (without holes at the bottom) (two plants per pot) containing full-strength Hoagland nutrient solution [31]. The nutrient solution was monitored regularly to maintain the pH and electrical conductivity. The solution was aerated by air pump to maintain proper root respiration. The salt-stress (12 dS m^−1^ NaCl) treatment was started 20 days after sowing (DAS) through a Hoagland nutrient solution. At the same time, calcium (Ca) in the form of CaSO_4_.5H_2_O (5, 10 and 15 mM) was sprayed on plant foliage every alternate day. The salinity level of nutrient solution was reported previously [20]. Salt stress and Ca treatment was maintained up to fruit maturity. Twenty-one days after salinity introduction (i.e., 41 DAS), tomato leaves were collected for different physiological and biochemical measurements. Control plants were provided with Hoagland’s nutrient solution only. Therefore, the treatment combinations were: control (T1) nutrient solution; 5 mM Ca (T2); 10 mM Ca (T3); 15 mM Ca (T4); 12 dS m^−1^ NaCl (T5); 12 dS m^−1^ NaCl + 5 mM Ca (T6); 12 dS m^−1^ NaCl + 10 mM Ca (T7); and 12 dS m^−1^ NaCl + 15 mM Ca (T8). The experimental pots were positioned in a completely randomized design with five replications. Plants were supported in perforated cork, and fitted into specially constructed metal covers.

### 2.2. Assessment of Growth and Yield Parameters

Crop harvesting was performed after nine weeks of treatment commencement. After harvesting, the plant samples were segmented into root, shoot, leaves and fruit. Plant height was measured by scale after harvest. The number of primary and secondary leaves of individual plant was counted. Shoot and root samples were oven dried at 70 °C for 72 h and then weighed. At the time of final harvest, the data on yield components, such as number of fruits per plant and fruit mass per plant, were recorded.

### 2.3. Estimation of Leaf Relative Water Content and Photosynthetic Pigment

After three weeks of treatment imposition, relative water content (RWC) of leaves was measured as described previously by Yamasaki and Dillenburg [32]. Twenty leaf discs (0.5 cm diameter) were collected from fully developed uppermost young leaves and their initial fresh weight recorded. Turgid weights were taken after keeping the discs in double-distilled water for 60 min. Dry weights were taken after oven drying at 70 °C for 72 h. The following formula was used for RWC calculation:

$$\mathrm{RWC}=\frac{\mathrm{Fresh}\;\mathrm{weight}-\mathrm{Dry}\;\mathrm{weight}}{\mathrm{Turgid}\;\mathrm{weight}-\mathrm{Dry}\;\mathrm{weight}}\times 100$$

Leaf chlorophyll concentration, known as SPAD (soil plant analysis development) value, was measured in upper three fully expanded leaves per plant with chlorophyll meter (SPAD-502, Minolta Co. Ltd., Tokyo, Japan). SPAD values were recorded at 30, 40, 50, 60, 70 and 80 days after sowing (DAS).

### 2.4. Determination of Proline and Glycine Betaine (GB) Content

Proline content was measured as described previously by Bates et al. [33]. A total of 500 mg fresh leaf sample was homogenized in sulfosalicylic acid and then centrifuged at 12,000× *g* for 10 min. After centrifugation, 2.0 mL of the supernatant were mixed with equal volumes of acid ninhydrin (25 mg/mL ninhydrin) and glacial acetic acid in a test tube. The test tubes were incubated at 100 °C for 1 h in a hot water bath. The slurry was placed on ice, and toluene was used to extract proline from samples before measuring the absorbance at 520 nm with a spectrophotometer (Shimadzu, UV-1201; 1, Nishinokyo Kuwabara-cho, Nakagyo-ku, Kyoto 604-8511, Japan). A standard curve was prepared with analytical-grade proline for calculating proline content expressed as mg g^−1^ fresh weight (FW) of leaves.

Glycine betaine content was determined according to the method of Grieve and Grattan [34]. A total of 500 mg dry leaf material was extracted with 20 mL of double-distilled water after shaking at room temperature for 24 h. A total of 2 N sulfuric acid was added to the filtered extract. Then, mixture of 0.5 mL aliquot and 0.2 mL cold potassium iodide was centrifuged at 10,000× *g* for 15 min. To dissolve the periodide-produced crystals, the supernatant was treated with 1,2-dichloroethane. After 3 h of the reaction, the absorbance was measured at 365 nm with a spectrophotometer (Shimadzu, UV-1201; 1, Nishinokyo Kuwabara-cho, Nakagyo-ku, Kyoto 604-8511, Japan). Standard reference curve was used for determination of GB content.

### 2.5. Determination of Hydrogen Peroxide and Melondealdehyde

Hydrogen peroxide (H_2_O_2_) was determined according to the method of Velikova et al. [35]. A total of 500 mg fresh leaf was macerated with 0.1% trichloroacetic acid (TCA), and the homogenate centrifuged at 10,000× *g* for 10 min. A total of 0.75 mL of 100 mM potassium phosphate buffer (pH 7.0) and 1 M potassium iodide were mixed with supernatant (0.75 mL). Spectrophotometer (Shimadzu, UV-1201; 1, Nishinokyo Kuwabara-cho, Nakagyo-ku, Kyoto 604-8511, Japan) was used to measure at 390 nm.

Malondialdehyde (MDA) was estimated as described previously by Madhava Rao and Sresty [36]. A total of 500 mg of fresh leaf were ground in 0.1% trichloroacetic acid (TCA) and centrifuged at 15,000× *g* for 10 min. Then, 1 mL of supernatant was added to 4 mL of thiobarbituric acid (TBA) (prepared in 20% TBA) and boiled at 100 °C for 30 min. The reaction mixture was terminated in an ice bath followed by centrifugation at 15,000× *g* for 10 min. Finally, the colored supernatants absorbance was measured at 530 nm and 600 nm using spectrophotometer (Shimadzu, UV-1201; 1, Nishinokyo Kuwabara-cho, Nakagyo-ku, Kyoto 604-8511, Japan).

### 2.6. Estimation of Antioxidant Enzymatic Activities

In the presence of 1 mL of ice-cold 100 mM potassium phosphate buffer (pH 7.0) containing 1% of polyvinyl pyrrolidone, the fresh leaf tissue was homogenized in a deep-freezer-cooled pestle and mortar. Supernatant, collected from the centrifuged (at 12,000× *g* for 30 min at 4 °C) homogenates was used to determine different enzyme activities. Catalase (CAT: 1.11.1.6) activity was determined according to the method of Aebi [37] through monitoring the decrease in absorbance at 240 nm for 1 min caused by the decomposition of H_2_O_2_ with a spectrophotometer (Shimadzu, UV-1201; 1, Nishinokyo Kuwabara-cho, Nakagyo-ku, Kyoto 604-8511, Japan). Ascorbate peroxidase (APX, 1.11.1.11) activity was determined according to Nakano and Asada [38]. The reaction mixture for the peroxidase contained 50 mM potassium phosphate, pH 7.0, 0.5 mM ascorbate, 0.1 mM hydrogen peroxide and 0.1 mM EDTA in a total volume of 1 mL. The H_2_O_2_-mediated oxidation of ascorbate was calculated from the decrease in absorbance at 290 nm min^−1^ when the extinction coefficient was 2.8 mM^−1^ cm^−1^ with a spectrophotometer (Shimadzu, UV-1201; 1, Nishinokyo Kuwabara-cho, Nakagyo-ku, Kyoto 604-8511, Japan).

### 2.7. Determination of Na, K, Ca, and Mg Contents

After harvest, tomato plants were separated into roots, stem and leaves, oven-dried separately at 70 °C for 72 h. Dried plant tissues (0.5 g) were digested with HNO_3_:HClO_4_ (5:1) acid mixture according to Rahman et al. [39]. From digested solution Na, K, Ca, and Mg contents were observed with atomic absorption spectrophotometer (Perkin-Elmer Analyst Model 2380, Waltham, MA, USA).

### 2.8. Estimation of Total Soluble Solids, Reducing Sugar, Total Sugar, β-Carotene, Ascorbic Acid (Vitamin C) and pH

Tomatoes taken from the same plant were cut into small pieces and mixed, constituting a sample. Then, the fruits were homogenized and used to determine total soluble solids (TSS), total and reducing sugars, β-Carotene, vitamin C and pH.

#### 2.8.1. Total Soluble Solids

Total soluble solids (%) contents of tomato fruit pulp were estimated with the help of refractometer (Model: Atago N1, Japan). A part of homogenized squeeze of tomato fruit was placed on the prism of the refractometer and the soluble solid contents were recorded as percent Brix.

#### 2.8.2. Estimation of Sugar

About 300 mg of tomato fruit was taken into liquid N_2_ and extracted in 0.7 M perchloric acid as described previously [40]. Starch in the insoluble fraction was determined by measuring the amount of glucose released by treatment with α-amylase and amyloglucosidase (both from Roche). Sugars (total sugars and reducing sugars) in the soluble fraction were determined using HPAEC-PAD (Dionex ICS-5000; Thermo Scientific; Waltham, MA, USA). Samples of the neutralized soluble fraction (200 μL) were applied to sequential 1.5 mL columns of cation exchanger Dowex 50 W and anion exchanger Dowex 1 (Sigma-Aldrich Canada Ltd. Oakville, ON, Canada). Neutral compounds were eluted with 5 mL of water, lyophilized, re-dissolved in 200 μL of water, and separated on a CarboPac PA20 column on an ICS-3000 system (Dionex; Sunnyvale, CA, USA) as previously described [41]. Peaks were identified by co-elution with known malto-oligosaccharide standards and areas were determined using the instrument’s Chromeleon software 7.2.

#### 2.8.3. Estimation of β-Carotene

One gram of sample was crushed and mixed thoroughly with 10 mL acetone–hexane (4:6) solution. This sample was centrifuged at 10,000 rpm for 15 min at 4 °C and optical density of the supernatant was measured using spectrophotometer (Shimadzu, UV-1201; 1, Nishinokyo Kuwabara-cho, Nakagyo-ku, Kyoto 604-8511, Japan) at 663 nm, 645 nm, 505 nm and 453 nm. Calculation was performed according to Nagata et al. [42].

#### 2.8.4. Estimation of Ascorbic Acid (Vit-C)

Ascorbic acid was determined as described previously [43]. A 0.2 g dry fruit sample was homogenized in 10 mL of 0.4% oxalic acid and the aqueous supernatant partitioned against chloroform, then diethyl ether, and, finally, filtered. Vitamin C (ascorbic acid) was determined at 245 nm and quantified against a standard solution of L-ascorbic acid (Sigma-Aldrich Canada Ltd. Oakville, ON, Canada) in 0.4% (*w*/*v*) oxalic acid.

#### 2.8.5. Fruit-Juice pH

To determine tomato fruit-juice pH, electrode of an electronic pH meter (Model no Inolab, pH-720; Blanchard Road, Burlington, Myanmar) was immersed in tomato juice for some time (1–2 min). Electronic pH meter was standardized with pH buffer solution before use and pH was recorded described previously [44].

### 2.9. Statistical Analysis

The observed data were evaluated statistically using ‘Statistix version 10’ software. The data were analyzed with analysis of variance (ANOVA) technique. Comparison of the mean difference was performed by least significant difference (LSD) test with a 5% level of significance.

## 3. Results

### 3.1. Growth and Biomass Production of Tomato

During sole application of calcium, the maximum plant height for BARI Tomato-2 (109 cm) and for Binatomato-5 (103 cm) was found in the 10 mM Ca-treated control plants (Table 1). Salinity reduced the plant height of both salt-tolerant BARI Tomato-2 and salt-susceptible Binatomato-5 by 30 and 39%, respectively, compared to the control plants. However, exogenous application of 5, 10 and 15 mM Ca to salinity-stressed plants enhanced plant height by 7%, 14%, and 5%, respectively for BARI Tomato-2 and 5%, 10%, and 3%, respectively, for Binatomato-5, compared to plant height treated with salinity alone (Table 1). The maximum number of leaves per plant^−1^ for BARI Tomato-2 (59) and for Binatomato-5 (56) was observed in the 10 mM Ca-treated control plants (Table 1). Salinity reduced leaves per plant^−1^ of BARI Tomato-2 and Binatomato-5 by 37% and 59%, respectively, compared to the control. During the combined application of salinity and Ca, statistically, the maximum number of leaves per plant^−1^ of BARI Tomato-2 (38) and Binatomato-5 (25) were observed in the T6 and T7 treatments.

It is apparent from Table 1 that without the application of Ca, salinity significantly reduced the shoot dry weight of tomato. Without salt application, the maximum shoot dry weight was found in the salt-tolerant BARI Tomato-2 (31.7 g) at T3 (10 mM Ca) (Table 1). Salinity reduced the shoot dry weight of both the salt-tolerant BARI Tomato-2 and salt-susceptible Binatomato-5 by 28.3 and 65%, respectively, compared to the control plants. However, the application of Ca^2+^ to 12 dS m^−1^ salinity significantly removed the salinity effect (Table 1). Following the combined application of NaCl and Ca, the maximum shoot dry weight was found in BARI Tomato-2 (25.8 g) at T7 (12 dSm^−1^ NaCl + 10 mM Ca). The root dry weight of both the salt-tolerant and salt-susceptible varieties showed a significant reduction under salinity conditions (Table 1). Following the application of only calcium, statistically, the maximum root dry weight was found in the T3 (1.7 g), T4 (1.7 g) and T2 (1.6 g) of BARI Tomato-2 and at T3 (1.7 g) of Binatomato-5 (Table 1). Salinity decreased the root dry weights of BARI Tomato-2 and Binatomato-5 by 44% and 68%, respectively, over control plants. However, exogenous application of 10 mM Ca to salinity-stressed plants enhanced root dry weight of BARI Tomato-2 and Binatomato-5 by 48% and 83%, respectively, compared to salinity alone.

### 3.2. Yield Components of Tomato

Number of fruits per plant^−1^ of tomato showed significant differences in response to different levels of calcium and salinity (Table 2). Table 2 shows that following the application of only Ca, the maximum number of fruits per plant^−1^ was found in BARI Tomato-2 (34) at T3 (10 mM Ca). Salinity reduced fruits per plant**^−1^** of BARI Tomato-2 and Binatomato-5 by 41% and 73%, respectively, compared to control. However, exogenous application of Ca to salinity-stressed plants further significantly increased fruits per plant^−1^. Consequently, following combined treatment, the maximum number of fruits per plant^−1^ was found in the BARI Tomato-2 (22) at T7 (12 dS m^−1^ NaCl + 10 mM Ca), which was 37% higher compared to salinity alone (T5). Without salt application, the maximum fruit mass per plant^−1^ was found in BARI Tomato-2 (2.32 kg) from 10 mM Ca (T3)-treated control plants (Table 2). Salinity significantly decreased the fruit mass of both salt-tolerant and salt-susceptible varieties. At 12 dS m^−1^ salinity, fruits mass per plant**^−1^** of BARI Tomato-2 and Binatomato-5 reduced by 25% and 61%, respectively, compared to the control. However, exogenous application of Ca to salinity-treated plants enhanced fruit mass per plant**^−1^**. Following combined treatment, statistically, the maximum fruit mass was found at T6 (1.55 kg) and T7 (1.58 kg) of BARI Tomato-2 (Table 2).

### 3.3. Relative Water Content and Chlorophyll Content (SPAD values) of Tomato

**Figure 1:**
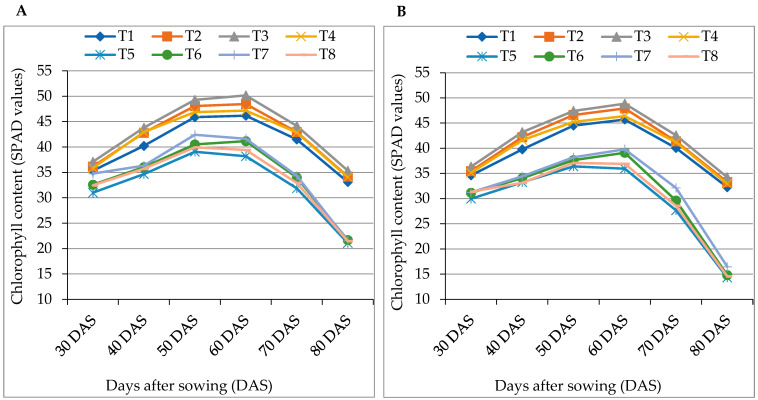
Foliar application of calcium regulates chlorophyll content (SPAD values) of (**A**) BARI Tomato-2 and (**B**) Binatomato-5 under salinity stress. T1 = control, T2 = 5 mM Ca, T3 = 10 mM Ca, T4 = 15 mM Ca, T5 = 12 dSm^−1^ NaCl, T6 = 12 dS m^−1^ NaCl + 5 mM Ca, T7 = 12 dS m^−1^ NaCl + 10 mM Ca, and T8 = 12 dS m^−1^ NaCl + 15 mM Ca.

Without salinity stress, statistically, the maximum relative water content (RWC) was found in T2 (87%), T3 (88%) and T4 (86%) of BARI Tomato-2 (Table 3). Under 12 dS m^−1^ salinity, the RWC of BARI Tomato-2 and Binatomato-5 reduced by 12% and 23%, respectively, compared to the control. Calcium exhibited better performance on RWC through mitigating the deleterious effect of salinity stress. During combined treatment, the highest RWC (82%) was found in BARI Tomato-2 at T7 (12 dS m^−1^ NaCl + 10 mM Ca), which was statistically dissimilar to other treatments (Table 3). The chlorophyll content (SPAD value) of leaves increased with the passage of time up to 60 days after sowing (DAS) and thereafter, declined gradually. Sole application of calcium significantly increased the formation of chlorophyll at different DAS (Figure 1A,B). At 60 DAS, the maximum SPAD value for BARI Tomato-2 (50.2) and for Binatomato-5 (48.8) was found in 10 mM Ca (T3)-treated control plants. Salinity significantly influenced the formation of chlorophyll as measured by the SPAD meter. Following the combined application of salinity and Ca, at 60 DAS, the maximum SPAD value for BARI Tomato-2 (41.5) and for Binatomato-5 (39.8) was observed at T7 (12 dS m^−1^ NaCl + 10 mM Ca).

### 3.4. Proline, Glycinebetaine, Hydrogen Peroxide and Malondialdehyde Content

**Figure 2:**
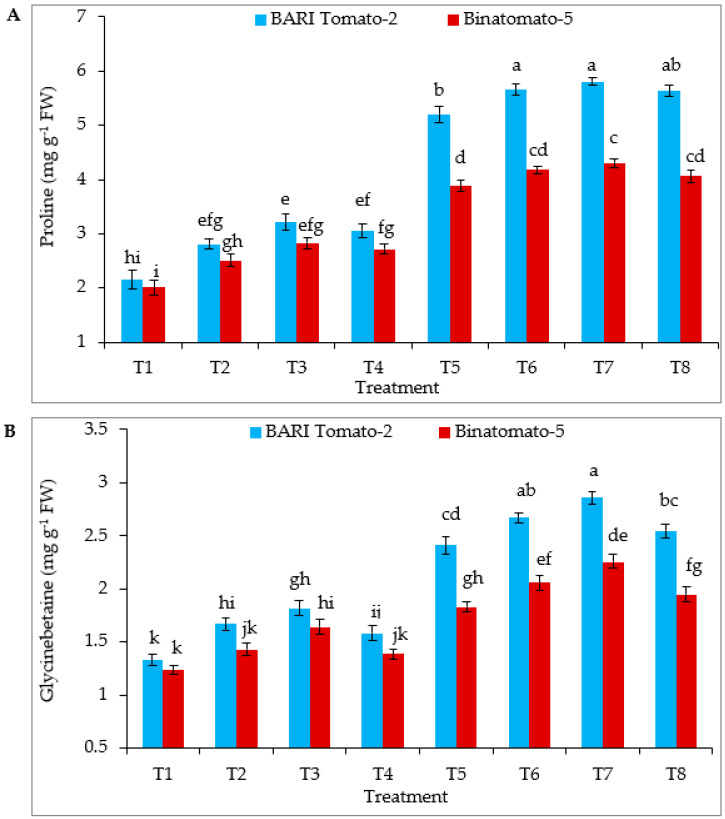
Foliar application of calcium regulates (**A**) proline and (**B**) glycinebetaine content of tomato leaves under salinity stress. The vertical bar indicates means of five replicates (n = 5) and the error bar indicates standard errors. Different letters indicate significant differences at *p* ≤ 0.05. T1 = control, T2 = 5 mM Ca, T3 = 10 mM Ca, T4 = 15 mM Ca, T5 = 12 dS m^−1^ NaCl, T6 = 12 dS m^−1^ NaCl + 5 mM Ca, T7 = 12 dS m^−1^ NaCl + 10 mM Ca, and T8 = 12 dS m^−1^ NaCl + 15 mM Ca.

In the absence of NaCl, statistically, the highest proline content was found in the T2, T3 and T4 of BARI Tomato-2, and T3 of Binatomato-5 (Figure 2A). Salinity increased the proline content of BARI Tomato-2 and Binatomato-5 by 142% and 93%, respectively, relative to the control. Exogenous application of Ca to salinity-treated plants further significantly increased proline content in both the varieties. However, the highest proline content was found in BARI Tomato-2 (5.81 mg g^−1^ FW) T7 (12 dS m^−1^ NaCl + 10 mM Ca), which was statistically similar to T6 and T8 of BARI Tomato-2. Following application of only calcium, statistically, the highest glycinebetaine (GB) content was found at T2, T3 of BARI Tomato-2, and T3 of Binatomato-5 (Figure 2B). Salinity significantly increased GB content in both the varieties. Exogenous application of Ca to salinity-stressed plants further significantly increased GB content in both the varieties; hence, statistically, the highest GB content was found in T6 and T7 of BARI Tomato-2.

**Figure 3:**
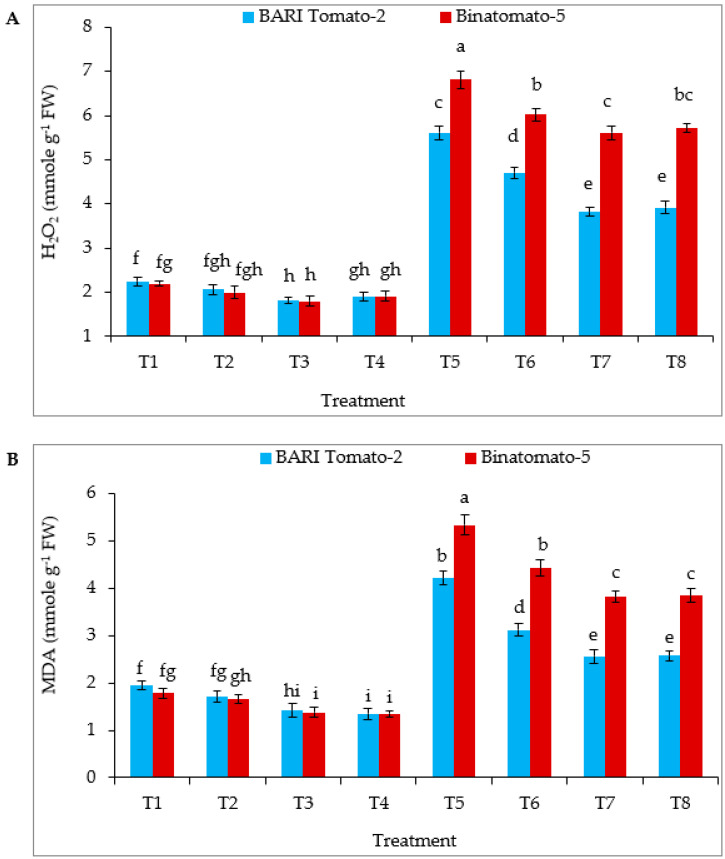
Foliar application of calcium regulates (**A**) H_2_O_2_ and (**B**) MDA content of tomato leaves under salinity stress. The vertical bar indicates means of five replicates (n = 5) and the error bar indicates standard errors. Different letters indicate significant differences at *p* ≤ 0.05. T1 = Control, T2 = 5 mM Ca, T3 = 10 mM Ca, T4 = 15 mM Ca, T5 = 12 dS m^−1^ NaCl, T6 = 12 dS m^−1^ NaCl + 5 mM Ca, T7 = 12 dS m^−1^ NaCl + 10 mM Ca, T8 = 12 dS m^−1^ NaCl + 15 mM Ca.

Figure 3 shows that salinity stress elevated hydrogen peroxide (H_2_O_2_) and malondialdehyde (MDA) accumulation in BARI Tomato-2 by 149 and 116% respectively, and in Binatomato-5 by 211 and 200%, respectively, relative to control. Exogenous application of Ca significantly decreased H_2_O_2_ and MDA accumulation in salinity-treated leaves compared to salinity alone in both the varieties. However, following combined treatment, statistically, the lowest H_2_O_2_ and MDA were found in the salinity-tolerant BARI Tomato-2 T7 and T8 treatments (Figure 3A,B).

### 3.5. Antioxidant Enzymes Activity

**Figure 4:**
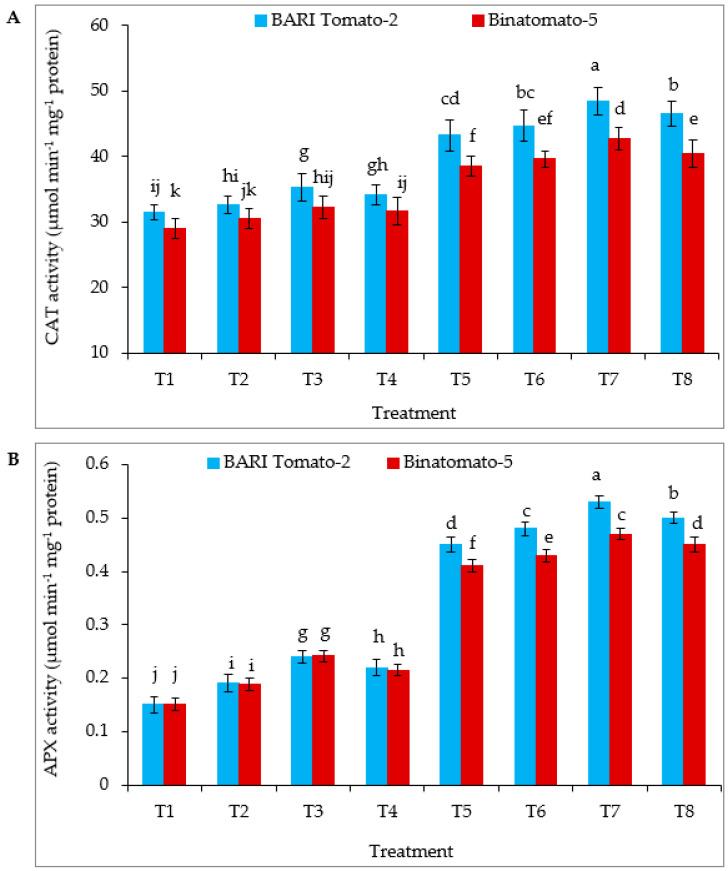
Foliar application of Ca enhanced the activity of (**A**) CAT, and (**B**) APX in two tomato varieties under salinity stress. The vertical bar indicates means of five replicates (n = 5) and the error bar indicates standard errors. Different letters indicate significant differences at *p* ≤ 0.05. T1 = control, T2 = 5 mM Ca, T3 = 10 mM Ca, T4 = 15 mM Ca, T5 = 12 dSm^−1^ NaCl, T6 = 12 dS m^−1^ NaCl + 5 mM Ca, T7 = 12 dS m^−1^ NaCl + 10 mM Ca, and T8 = 12 dS m^−1^ NaCl + 15 mM Ca.

Salinity-stress-elevated catalase (CAT) and ascorbate peroxidase (APX) activities increased in BARI Tomato-2 by 37 and 200%, respectively, and in Binatomato-5 by 33 and 171% respectively, relative to control (Figure 4A,B). Exogenous application of Ca further increased CAT and APX activities in salinity-treated leaves compared to salinity alone in both the varieties. However, among all the treatment, the highest CAT (48.42 µmol min^−1^ mg^−1^ protein) and APX (0.53 µmol min^−1^ mg^−1^ protein) activities were found in BARI Tomato-2 T7 (12 dS m^−1^ NaCl + 10 mM Ca) treatment.

### 3.6. Mineral Concentrations

Salinity stress significantly increased leaves and root sodium (Na^+^) concentration and Na^+^/K^+^ ratio compared to the control (Table 4). On the other hand, salinity-treated plants supplemented with Ca significantly decreased leaves and root Na^+^ concentration and Na^+^/K^+^ ratio (Table 4). Among all the treatments, the highest leaves and root Na^+^ (0.69% and 0.90%, respectively) was observed in Binatomato-5 T5 (12 dS m^−1^ NaCl). Following sole application of Ca, statistically, the highest potassium (K^+^) in leaves was found in the T1, T2, T3, T4 treatments, and root potassium (K^+^) was found in the T3 and T4 treatments of Binatomato-5. However, statistically, the highest leaves and root calcium (Ca^2+^) (3.05% and 4.31%, respectively) and magnesium (Mg^2+^) (0.93% and 0.60%, respectively) was found in the T3 of BARI Tomato-2. Nevertheless, leaves and root concentration of K^+^, Ca^2+^ and Mg^2+^ was significantly reduced by salinity treatment compared to control plants, whereas salinity-treated plants supplemented with Ca significantly increased leaves and root K^+^, Ca^2+^ and Mg^2+^ concentration (Table 4).

### 3.7. Fruit-Quality Traits of Tomato

Salinity stress significantly increased total soluble solid (TSS) content of tomato fruits compared to the control (Table 5). On the other hand, salinity-treated plants supplemented with exogenous Ca further significantly increased the TSS content of both the varieties (Table 5). Among all the treatments, the highest TSS (7.78%) was observed in BARI Tomato-2 T7 (12 dS m^−1^ NaCl + 10 mM Ca). Following application of only Ca, the maximum total sugar (TS), reducing sugar (RS) and β-carotene was found in BARI Tomato-2 (3.95%, 2.86%, 0.196 mg/100 g FW, respectively) from 10 mM Ca (T3)-treated control plants (Table 5). However, TS, RS and β-carotene content was significantly increased by salinity treatment compared to control plants, while salinity-treated plants supplemented with exogenous Ca further significantly increased the TS, RS and β-carotene content of fruits of both the varieties (Table 5). During combined treatment, the maximum TS, RS and β-carotene of tomato fruit was found in the BARI Tomato-2 (5.01%, 3.41% and 0.274 mg/100 g FW, respectively) T7 (12 dS m^−1^ NaCl + 10 mM Ca) treatment. Following combined treatment, statistically, the maximum TS was found in T7; RS was found in T5, T6, T7; and β-carotene was found in the T6 and T7 of BARI Tomato-2.

Regarding absence of salinity, statistically, the maximum vitamin C was found in T2- and T3-treated control plants of both the tomato varieties. Salinity significantly increased the vitamin C content of both the tomato varieties. However, during combined treatment, statistically, the maximum vitamin C was found in T6 and T7 of BARI Tomato-2. Salinity and calcium have no significant effect on the tomato-juice pH of both the varieties (Table 5). In the absence of salinity, the maximum juice pH was found in BARI Tomato-2 (4.55) from 10 mM Ca (T3)-treated control plants. Salinity reduced the juice pH of BARI Tomato-2 and Binatomato-5 by 2.9% and 5.4%, respectively, relative to the control. When exogenous Ca was applied to salinity-stressed plants, the maximum juice pH was found in BARI Tomato-2 (4.54) at T7 (12 dSm^−1^ NaCl + 10 mM Ca).

## 4. Discussion

### 4.1. Growth and Biomass Production of Tomato

Salinity stress significantly reduced the plant height, leaves per plant^−1^ and biomass yield (shoot and root dry weight) of tomato plants (Table 1). Reduction in plant growth and biomass yield is a common impact of salinity in several plant species [45,46,47]. These reductions might be due to the stress-forced inhibition of cell elongation, cell division, and inhibition of nutrient uptake to plants. The ionic imbalance in the tomato plant due to excess amounts of salt also might be the reason for the reduction in the growth parameters of the tomato plants. It was reported that saline-induced disturbance of ion homeostasis, and osmotic and oxidative status cause growth reduction [39]. Under salinity conditions, the uptake of Na^+^ was generally high compared to K^+^ and Ca^2+^, thus inducing ion deficiencies (Table 4; [5]). Salinity establishes a water potential imbalance between the apoplast and symplast which leads to turgor decrease, which leads to growth reduction. It was reported that inhibition of crop growth under salt stress was reduced through exogenous application of Ca [23,48,49]. Exogenous Ca application also might enhance the uptake of mineral elements and, hence, reduce the effects of cadmium stress on the growth and yield of crops [50].

### 4.2. Yield Components of Tomato

Salinity reduced the number of fruits per plant^−1^ and ultimately reduced the fruit yield of tomato (Table 2), which is supported by Parvin et al. [49] and Khanbabaloo et al. [51]. Salt stress enhances programmed cell death and inhibits micro-sporogenesis and stamen filament elongation. It also increases ovule abortion and senescence of fertilized embryo and, finally, affects reproductive development [52]. It was stated that photosynthesis, nutrient uptake, water absorption, root growth, and cellular metabolism were severely affected due to salt stress, which leads to yield reduction [23]. Salinity stress reduced the fruits per plant**^−1^** of BARI Tomato-2 and Binatomato-5 by 41.0% and 72.5%, respectively, and the fruit yield of BARI Tomato-2 and Binatomato-5 by 24.6% and 60.1%, respectively, compared to the control (Table 2). It was reported that a reduction in grain yield due to salinity compared to control conditions is used as an indicator of tolerance to salinity stress [53]. Statistically significant variation was recorded for the number of fruits per plant^−1^ and fruit yield of tomato due to the application of different levels of calcium (Table 2). These results suggested that calcium reduced the toxic effect of salinity and increased the fruit yield of tomato, which agrees with the result of [49,54]. Park et al. [55] stated that calcium deficiency affects tomato fruit development. Calcium participates both in the alleviation of sodium toxicity and in the fruit-size development [27,56]. Calcium increases the fruit yield of tomato through leaf-size enlargement and protection of young leaves from necrosis [54].

### 4.3. Relative Water Content and Chlorophyll Content (SPAD Values) of Tomato

Salinity stress reduced the relative water content (RWC) of BARI Tomato-2 and Binatomato-5 by 12.2% and 23.0%, respectively, compared to the control (Table 3). This reduction might be due to salt-induced constraints on the availability and uptake of water and injury to root system of tomato. Due to an imbalance in osmotic pressure, plants suffer from osmotic stress, which makes the root too rigid to uptake water under salt stress. Salt stress reduces the root water potential by accumulating salt in root zones and restricts water uptake, which may cause osmotic imbalances [5,10]. The exogenous application of Ca^2+^ had a positive effect on RWC in both tomato varieties under salt stress, which is likely due to reducing membrane injury and improving the water balance. Calcium might positively affect stomatal functions by keeping the guard cells turgid [57]. Excess Na^+^ ion depolarizes root plasma membranes which activate guard cells outward, rectifying potassium channels, decreasing cytosolic K^+^, displacing Ca^2+^ from membranes, and, consequently, disrupting the ion homeostasis [58,59,60]. In contrast, exogenous application of Ca^2+^ reduces salt toxicity by decreasing Na^+^ content through reducing its uptake and transport and preventing its binding to cell walls. It also assists water and mineral uptake and promotes membrane stability [39].

The photosynthetic potential of plants reflects their overall performance, which is expressed using various biomass and growth parameters. Salt stress significantly decreased photosynthetic pigments such as chlorophyll content (SPAD value) at different growth stages of tomato, while exogenous application of Ca on salinity-stressed tomato significantly increased the chlorophyll (SPAD value) content (Figure 1A,B). Salt stress accelerates the activity of chlorophyllase through destabilizing the pigments associated with the chlorophyll protein complex and reducing the amount of photosynthetic pigments [3]. The accumulation of Na^+^ and Cl^−^ ions increases due to salinity stress, which hampers the process of chlorophyll synthesis by influencing the activity of some chlorophyll synthesizing enzymes containing Fe^3+^ [61]. On the contrary, exogenous Ca increases the activity of enzymes associated with the carbon reactions of photosynthesis by reducing the uptake of Na^+^ ions. Thus, salt-stress-induced inhibition of photosynthetic capacity is reduced by Ca. These findings are also consistent with those of Hayat et al. [62] in *Vigna radiata*, Ahmad et al. [50] in *Brassica juncea* and Roy et al. [23] in *Oryza sativa*. It was also reported that Ca enhances cytokinin action, which increases chlorophyll synthesis [63].

### 4.4. Biochemical Traits of Tomato

To combat the negative effects of salinity stress, plants trigger the production of osmolytic cytosolutes, which alleviates physiological damage [52,64]. In the present study, salinity stress significantly increased the proline and glycinebetaine (GB) content, and these levels increased further with the exogenous application of Ca (Figure 2A,B). Under salinity conditions, the accumulation of proline and GB show an increasing trend, helping in cell osmoregulation and alleviating salt stress in different plant species [23,65,66]. Proline and GB buffer the photosynthetic machinery and act as molecular chaperones, provide energy storage, protect membranes and enzyme activity, and modulate gene activation related to stress [67,68,69]. Both of these osmolytes reduce oxidative stress by increasing the activities of those enzymes associated with the scavenging of reactive oxygen species (ROS) and malondialdehyde (MDA) and also by neutralizing the effects of those free radicals [67,69]. Moreover, proline and GB also restore photosynthetic efficiency and photoassimilate production and, hence, plant growth and productivity by regulating salt-stress-mediated oxidative stress [12,69] It was observed that GB also prevents the inactivation of Rubisco and the oxygen-evolving complex of PSII [70]. It was reported that Ca is an important signaling molecule which is involved in proline biosynthesis [71].

Accumulation of excess amounts of ROS and MDA are indicators of oxidative stress under salt stress [12,72]. In the present study, salinity significantly increased the accumulation of H_2_O_2_ and MDA, and exogenous application of Ca significantly decreased their accumulation in the salinity-treated plants (Figure 3). ROS and MDA hamper cell-membrane integrity in plants, which, consequently, increases electrolyte leakage [3,39]. Furthermore, salt-treated higher root electrolyte leakage might be the reason for root damage and consequent osmotic suffering. However, exogenous application of Ca in salt-stressed tomato alleviated ROS and MDA accumulation and reduced oxidative damage in tomato (Figure 3A,B). It was reported that Ca bonds to the phospholipid bilayer of cellular membranes, thus stabilizing the lipid bilayer and providing structural integrity during stress [73].

### 4.5. Antioxidant Enzymes Activity

A variety of stresses induce the production of ROS in plants. Under stress, plants activate enzymatic and non-enzymatic antioxidants, which regulate oxidation stress and protect plant cells from oxidative damage by scavenging ROS [74]. The present study revealed that APX and CAT activity increased in both tolerant and susceptible tomato varieties in response to salinity stress (Figure 4A,B), which is a similar response to other species such as *Oryza sativa* [23], *Brassica juncea* [50], *Glycine max* [75]. It is recognized that CAT involves the removal of overproduced H_2_O_2_ during oxidative stress [76]. CAT could convert hydrogen peroxide into oxygen and water to remove the peroxide in plants, and the higher action of CAT led to greater salt tolerance [77]. The ascorbate–glutathione cycle is a major H_2_O_2_ detoxifying system in plant cells, in which APX enzymes play a key role in catalyzing the conversion of H_2_O_2_ into H_2_ using ascorbate as a specific electron donor [78]. In the present study, exogenous application of Ca increased antioxidant enzyme activity (Figure 4), which is consistent with Roy et al. [23] under salinity stress.

### 4.6. Mineral Concentrations

In the present study, salt stress significantly increased the uptake of Na^+^ in leaves and roots, while the uptake of K^+^, Ca^2+^, and Mg^2+^ were reduced drastically (Table 4). Under salt stress, a large electrochemical gradient of ions occured because the rhizosphere is surrounded by Na^+^ ions, resulting in an influx of Na^+^ ions via membrane-located channels and transporters on the plasma membrane [60,79]. An antithetical relationship between Na^+^ and other essential minerals ions, such as K^+^, Ca^2+^ and Mg^2+^, alters Ca^2+^, Mg^2+^ and the ratios of Na^+^/K^+^ and, finally, creates ion imbalance under salt stress. In the present study, we observed that an influx of excess Na^+^ ions, which raised their endogenous concentration to trigger K^+^ efflux, was reflected in the low K^+^ content and high Na^+^/K^+^ ratio both in leaves and roots, resulting in disturbed ion homeostasis which may displace Ca^2+^ by Na^+^. Hossain et al. [18] stated that the excess influx of Na^+^ in roots reduces the uptake, transportation, and accumulation of other essential minerals including K^+^, Ca^2+^, and Mg^2+^, and causes nutrient starvation. The Na^+^ ion influx into the cell and other mineral ion leakage from the cell may also lead to higher ROS accumulation. However, an adequate amount of K^+^, Ca^2+^, and Mg^2+^ in plants are essential for basic metabolic processes such as intracellular K^+^ homeostasis, which is essential for optimal functioning of photosynthetic machinery and maintenance of stomatal opening [80]. A sufficient amount of K^+^, Ca^2+^, and Mg^2+^ are also essential for various enzymatic activities, and a deficiency of these elements will inhibit protein synthesis and reduce crop growth [81]. Exogenous application of Ca inhibits influx of Na^+^ ion; efflux of Ca^2+^, Mg^2+^ and K^+^; and reduces Na^+^/K^+^ ratio, thereby conferring salinity tolerance by regulating ROS production and improving photosynthesis [26].

### 4.7. Fruit Quality of Tomato

Total soluble solids (TSS), total sugar, reducing sugar, and β-carotene content were significantly influenced by salinity and various levels of Ca alone or in combination (Table 5). These findings are consistent with many other researchers [20,51,54,56,82,83,84]. Salinity has positively improved the TSS and other organic compounds, which might be due to increased accumulation of Na^+^, K^+^ and chorine (Cl^−^) ions in fruit [56]. K^+^ concentration in the fruit has been shown to be positively correlated with carbohydrate metabolism [20,56]. Foliar application of Ca increases the TSS, total sugar, and reducing sugar of tomato, which might be due to a higher accumulation of metabolites and the immediate alteration of starch into soluble sugar during fruit growth and development in response to growth regulators. It was reported that enzymes associated with sugar biosynthesis might be regulated by Ca and salinity [85]. Enhancement of TSS might be due to the conversion of starch, protein, pectin and hemicelluloses into simple soluble sugars and reduction in the water content of fruit [86].

Vitamin C is a ubiquitous molecule for improving abiotic and biotic stress tolerance in plants [86]. It can improve tolerance to abiotic stresses by enhancing plant growth, photosynthetic pigments, photosynthesis rate, transpiration and oxidative defense potential [87]. Table 5 showed that salinity stress significantly increased the vitamin C content, and these levels increased further with the supplementation of Ca. Vitamin C concentration increases as a response to abiotic stress through de-novo synthesis or due to its regeneration from dihydrolipoic acid [88]. Higher TSS and vitamin C may explain the effect of the Ca^2+^ on the metabolism of soluble solids and organic acids [54,82]. It is also reported that vitamin C biosynthesis generally parallels a high level of sugar content [82]. Tomato fruit-juice pH was not significantly influenced by salinity and various levels of Ca. However, salinity slightly decreases the juice pH, and supplementation of Ca slightly increased the juice pH in the salinity-treated plants (Table 5). These findings are consistent with those of Botella et al. [20] and Sangtarashani et al. [54], who reported that salinity or a combination of salinity with Ca does not significantly increase the fruit-juice pH of tomato.

## 5. Conclusions

In the present study, we investigated the exogenous Ca-induced salinity-stress tolerance in tomato cultivars. This study revealed that both the cultivars suffered from salt exposure; however, the susceptible one suffered more in terms of growth, fruit yield and chlorophyll reduction, as well as osmotic and ionic stress, than the tolerant one. Salt exposure also increases the production of ROS, which causes lipid peroxidation (MDA). However, exogenous application of Ca enhanced growth, yield and chlorophyll content. Ca application reduced oxidative stress through regulating the antioxidant defense and ROS detoxification system by boosting antioxidant enzymatic activity. Exogenous Ca application in salt-treated plants induced proline and glycine betaine content, uptake of K^+^, Ca^2+^, and Mg^2+^ and reduced Na^+^ uptake, which resulted in the inhibition of ionic toxicity and osmotic stress, respectively. Calcium application also enhanced the fruit quality of tomato. Our results demonstrate that the application of Ca reversed the negative impact of salinity stress through the modulation of physiological attributes, biochemical parameters, and enzymatic activities of antioxidants. Therefore, further comprehensive research is essential to explore endogenous Ca synthesis along with mineral homeostasis and signaling approaches for higher osmoregulation with antioxidant defense, which is vital to future crop productivity.

## Figures and Tables

**Table 1:** Foliar application of calcium-enhanced growth and biomass production of tomato under salinity stress.

Variety	Treatment		Plant Height (cm)	Leaves per plant^−1^	Shoot Dry Weight (g per plant^−1^)	Root Dry Weight (g per plant^−1^)
BARI Tomato-2	Control	T1	103 ± 1.97 c	55 ± 2.08 bc	27.7 ± 0.21 d	1.5 ± 0.09 cd
	5 mM Ca	T2	107 ± 2.89 ab	56 ± 1.53 b	29.5 ± 0.70 b	1.6 ± 0.10 abc
	10 mM Ca	T3	109 ± 3.06 a	59 ± 2.08 a	31.7 ± 0.63 a	1.7 ± 0.13 a
	15 mM Ca	T4	103 ± 2.52 bc	55 ± 1.73 bc	29.8 ± 0.60 b	1.7 ± 0.17 ab
	12 dS m^−1^ NaCl	T5	72 ± 2.45 g	34 ± 2.52 g	19.9 ± 0.65 i	0.9 ± 0.07 g
	T5 + 5 mM Ca	T6	77 ± 3.09 f	37 ± 1.53 ef	22.7 ± 0.25 g	1.0 ± 0.12 f
	T5 + 10 mM Ca	T7	81 ± 2.48 e	38 ± 1.53 e	25.8 ± 0.15 f	1.3 ± 0.09 e
	T5 + 15 mM Ca	T8	75 ± 1.79 f	36 ± 1.53 fg	21.4 ± 0.65 h	1.1 ± 0.08 f
Binatomato-5	Control	T1	98 ± 2.99 d	51 ± 2.08 d	26.7 ± 0.07 e	1.5 ± 0.09 d
	5 mM Ca	T2	102 ± 2.09 c	55 ± 1.73 bc	28.4 ± 0.96 cd	1.5 ± 0.13 cd
	10 mM Ca	T3	103 ± 2.03 bc	56 ± 1.15 b	29.6 ± 0.89 b	1.7 ± 0.14 ab
	15 mM Ca	T4	100 ± 2.78 cd	53 ± 1.63 cd	28.5 ± 0.33 c	1.6 ± 0.15 bcd
	12 dS m^−1^ NaCl	T5	59 ± 1.66 i	21 ± 0.72 i	9.5 ± 0.13 l	0.5 ± 0.06 i
	T5 + 5 mM Ca	T6	62 ± 1.67 hi	24 ± 1.15 h	11.1 ± 0.51 k	0.6 ± 0.08 h
	T5 + 10 mM Ca	T7	65 ± 2.65 h	25 ± 0.90 h	13.6 ± 0.56 j	0.9 ± 0.12 g
	T5 + 15 mM Ca	T8	61 ± 1.75 i	23 ± 0.35 hi	10.3 ± 0.31 k	0.8 ± 0.10 gh
CV (%)			2.51	3.26	2.03	6.44
LSD_(0.05)_			3.61	2.31	0.77	0.133

Mean values followed by different letters in the same column are significantly different from each other at 5% level of significance. Means of five replicates are shown ± SD. CV: coefficient of variation; LSD: least significant difference at 5% level; SD: standard deviation.

**Table 2:** Foliar application of calcium-enhanced fruits number and fruit yield of tomato under salinity stress.

Variety	Treatment		Number of Fruits per Plant^−1^	Fruits Mass (kg) per Plant^−1^
BARI Tomato-2	Control	T1	28 ± 0.84 f	1.71 ± 0.19 c
	5 mM Ca	T2	31 ± 1.03 c	2.09 ± 0.29 b
	10 mM Ca	T3	34 ± 1.06 a	2.32 ± 0.26 a
	15 mM Ca	T4	31 ± 0.97 d	2.05 ± 0.16 b
	12 dS m^−1^ NaCl	T5	16 ± 0.69 j	1.29 ± 0.22 f
	T5 + 5 mM Ca	T6	21 ± 0.61 h	1.55 ± 0.14 cd
	T5 + 10 mM Ca	T7	22 ± 0.85 g	1.58 ± 0.14 cd
	T5 + 15 mM Ca	T8	19 ± 0.41 i	1.37 ± 0.15 ef
Binatomato-5	Control	T1	28 ± 0.67 f	1.53 ± 0.16 de
	5 mM Ca	T2	30 ± 1.05 e	1.71 ± 0.12 c
	10 mM Ca	T3	32 ± 1.17 b	1.98 ± 0.12 b
	15 mM Ca	T4	30 ± 0.93 e	1.64 ± 0.21 cd
	12 dS m^−1^ NaCl	T5	8 ± 0.44 n	0.61 ± 0.18 h
	T5 + 5 mM Ca	T6	11 ± 0.56 l	0.84 ± 0.11 g
	T5 + 10 mM Ca	T7	12 ± 0.41 k	0.89 ± 0.10 g
	T5 + 15 mM Ca	T8	10 ± 0.66 m	0.77 ± 0.06 gh
CV (%)			1.51	7.03
LSD_(0.05)_			0.57	0.175

Mean values followed by different letters in the same column are significantly different from each other at 5% level of significance. Means of five replicates are shown ± SD. CV: coefficient of variation; LSD: least significant difference at 5% level; SD: standard deviation.

**Table 3:** Foliar application of calcium-enhanced relative water content of tomato under salinity stress.

Variety	Treatment		Relative Water Content (%)
BARI Tomato-2	Control	T1	85 ± 1.80 bcd
	5 mM Ca	T2	87 ± 2.21 ab
	10 mM Ca	T3	88 ± 1.53 a
	15 mM Ca	T4	86 ± 2.12 abc
	12 dS m^−1^ NaCl	T5	75 ± 2.14 g
	T5 + 5 mM Ca	T6	79 ± 1.75 f
	T5 + 10 mM Ca	T7	82 ± 2.18 e
	T5 + 15 mM Ca	T8	76 ± 1.45 g
Binatomato-5	Control	T1	84 ± 1.19 d
	5 mM Ca	T2	85 ± 1.11 bcd
	10 mM Ca	T3	86 ± 1.65 bc
	15 mM Ca	T4	85 ± 0.66 cd
	12 dS m^−1^ NaCl	T5	65 ± 1.35 j
	T5 + 5 mM Ca	T6	69 ± 0.58 i
	T5 + 10 mM Ca	T7	71 ± 0.94 h
	T5 + 15 mM Ca	T8	65 ± 1.35 j
CV (%)			1.34
LSD_(0.05)_			1.76

Mean values followed by different letters in the same column are significantly different from each other at 5% level of significance. Means of five replicates are shown ± SD. CV: coefficient of variation; LSD: least significant difference at 5% level; SD: standard deviations.

**Table 4:** Foliar application of calcium regulates leaves and roots’ nutrient concentrations of tomato under salinity stress.

Variety	Treatment		%Na^+^		%K^+^		Na ^+^ /K^+^		%Ca^2+^		%Mg^2+^	
			Leaves	Roots	Leaves	Roots	Leaves	Roots	Leaves	Roots	Leaves	Roots
BARI Tomato-2	Control	T1	0.26 ± 0.02 gh	0.51 ± 0.01 ef	2.73 ± 0.08 b	1.37 ± 0.02 f	0.10 ± 0.01 g	0.37 ± 0.01 h	2.37 ± 0.02 f	3.56 ± 0.01 f	0.79 ± 0.03 efg	0.53 ± 0.02 bc
	5 mM Ca	T2	0.23 ± 0.01 ij	0.42 ± 0.03 hi	2.77 ± 0.05 b	1.52 ± 0.01 e	0.08 ± 0.00 ghi	0.28 ± 0.02 i	2.79 ± 0.05 c	3.91 ± 0.02 c	0.84 ± 0.03 cd	0.53 ± 0.09 bc
	10 mM Ca	T3	0.22 ± 0.01 j	0.38 ± 0.01 i	2.77 ± 0.05 b	1.63 ± 0.05 c	0.08 ± 0.00 ghi	0.24 ± 0.01 ijk	3.05 ± 0.06 a	4.31 ± 0.04 a	0.93 ± 0.02 a	0.60 ± 0.00 a
	15 mM Ca	T4	0.23 ± 0.01 hij	0.39 ± 0.01 i	2.76 ± 0.05 b	1.59 ± 0.04 d	0.08 ± 0.00 ghi	0.24 ± 0.01 ij	2.89 ± 0.02 b	4.19 ± 0.02 b	0.89 ± 0.02 ab	0.56 ± 0.04 ab
	12 dS m^−1^ NaCl	T5	0.56 ± 0.07 bc	0.68 ± 0.05 c	2.13 ± 0.06 f	0.83 ± 0.01 i	0.26 ± 0.03 b	0.82 ± 0.07 d	2.05 ± 0.08 i	2.89 ± 0.01 k	0.65 ± 0.01 i	0.39 ± 0.00 fgh
	T5 + 5 mM Ca	T6	0.46 ± 0.01 e	0.61 ± 0.06 d	2.23 ± 0.02 def	0.87 ± 0.03 hi	0.20 ± 0.00 d	0.71 ± 0.09 e	2.27 ± 0.05 g	3.03 ± 0.02 j	0.75 ± 0.01 gh	0.44 ± 0.00 ef
	T5 + 10 mM Ca	T7	0.37 ± 0.01 f	0.50 ± 0.01 fg	2.41 ± 0.03 c	0.97 ± 0.01 g	0.15 ± 0.01 f	0.52 ± 0.01 g	2.50 ± 0.04 e	3.25 ± 0.02 h	0.77 ± 0.00 efg	0.47 ± 0.01 de
	T5 + 15 mM Ca	T8	0.43 ± 0.01 e	0.57 ± 0.02 de	2.33 ± 0.04 cde	0.89 ± 0.01 h	0.18 ± 0.00 e	0.63 ± 0.03 f	2.10 ± 0.01 hi	2.91 ± 0.05 k	0.72 ± 0.00 h	0.42 ± 0.01 efg
Binatomato-5	Control	T1	0.29 ± 0.00 g	0.45 ± 0.07 gh	3.14 ± 0.02 a	1.53 ± 0.02 e	0.09 ± 0.00 gh	0.30 ± 0.04 i	2.15 ± 0.00 h	3.11 ± 0.00 i	0.76 ± 0.05 fgh	0.50 ± 0.04 cd
	5 mM Ca	T2	0.25 ± 0.01 hij	0.37 ± 0.02 ij	3.18 ± 0.07 a	1.78 ± 0.04 b	0.08 ± 0.00 hi	0.21 ± 0.01 jkl	2.57 ± 0.06 d	3.46 ± 0.02 g	0.81 ± 0.02 def	0.51 ± 0.00 bcd
	10 mM Ca	T3	0.23 ± 0.01 ij	0.31 ± 0.01 j	3.18 ± 0.01 a	1.86 ± 0.02 a	0.07 ± 0.00 i	0.17 ± 0.01 l	2.82 ± 0.08 bc	3.83 ± 0.01 d	0.86 ± 0.03 bc	0.56 ± 0.01 ab
	15 mM Ca	T4	0.26 ± 0.01 ghi	0.32 ± 0.03 j	3.18 ± 0.02 a	1.83 ± 0.04 a	0.08 ± 0.01 ghi	0.18 ± 0.02 kl	2.64 ± 0.05 d	3.68 ± 0.07 e	0.81 ± 0.03 cde	0.53 ± 0.01 bc
	12 dS m^−1^ NaCl	T5	0.69 ± 0.02 a	0.90 ± 0.02 a	2.10 ± 0.5 f	0.74 ± 0.01 l	0.33 ± 0.03 a	1.23 ± 0.03 a	1.30 ± 0.02 l	1.87 ± 0.01 o	0.44 ± 0.01 k	0.31 ± 0.00 j
	T5 + 5 mM Ca	T6	0.57 ± 0.01 b	0.84 ± 0.02 b	2.19 ± 0.01 ef	0.76 ± 0.01 j	0.26 ± 0.00 b	1.11 ± 0.03 b	1.64 ± 0.02 k	1.98 ± 0.01 n	0.58 ± 0.00 j	0.36 ± 0.00 hij
	T5 + 10 mM Ca	T7	0.50 ± 0.01 d	0.70 ± 0.05 c	2.34 ± 0.04 cd	0.83 ± 0.01 i	0.21 ± 0.01 d	0.84 ± 0.05 d	1.73 ± 0.02 j	2.37 ± 0.03 l	0.61 ± 0.00 ij	0.38 ± 0.01 ghi
	T5 + 15 mM Ca	T8	0.53 ± 0.03 c	0.71 ± 0.05 c	2.23 ± 0.05 def	0.76 ± 0.00 j	0.24 ± 0.01 c	0.94 ± 0.07 c	1.66 ± 0.02 jk	2.16 ± 0.03 m	0.58 ± 0.00 j	0.33 ± 0.01 ij
CV (%)			5.67	6.34	3.32	1.94	7.20	7.19	1.98	0.89	3.87	7.06
LSD_(0.05)_			0.036	0.057	0.144	0.040	0.019	0.066	0.075	0.047	0.048	0.055

Mean values followed by different letters in the same column are significantly different from each other at 5% level of significance. Means of five replicates are shown. CV: coefficient of variation; LSD: least significant difference at 5% level; SD: standard deviation.

**Table 5:** Foliar application of calcium regulates total soluble solids (TSS, Brix), total sugar, reducing sugar, beta-carotene, vitamin C content and juice pH of tomato fruit under salinity stress.

Variety	Treatment		TSS (%)	Total Sugar (%)	Reducing Sugar (%)	β-Carotene (mg/100 g FW)	Vitamin C (mg/100 g FW)	Juice pH
BARI Tomato-2	Control	T1	5.57 ± 0.55 gh	3.84 ± 0.13 f	2.76 ± 0.19 bc	0.181 ± 0.00 hi	18.00 ± 0.44 i	4.50 ± 0.33
	5 mM Ca	T2	5.62 ± 0.55 gh	3.85 ± 0.13 f	2.78 ± 0.15 bc	0.191 ± 0.01 fg	19.78 ± 0.38 g	4.50 ± 0.55
	10 mM Ca	T3	5.75 ± 0.55 fg	3.95 ± 0.13 e	2.86 ± 0.15 b	0.196 ± 0.01 f	20.03 ± 0.11 fg	4.55 ± 0.55
	15 mM Ca	T4	5.50 ± 0.51 h	3.72 ± 0.08 g	2.69 ± 0.05 bcd	0.184 ± 0.01 gh	18.71 ± 0.09 h	4.53 ± 0.55
	12 dS m^−1^ NaCl	T5	7.21 ± 0.55 c	4.61 ± 0.11 c	3.18 ± 0.20 a	0.251 ± 0.01 b	22.15 ± 0.54 bc	4.37 ± 0.23
	T5 + 5 mM Ca	T6	7.51 ± 0.55 b	4.74 ± 0.07 b	3.25 ± 0.20 a	0.260 ± 0.01 b	22.51 ± 0.14 ab	4.48 ± 0.07
	T5 + 10 mM Ca	T7	7.78 ± 0.25 a	5.01 ± 0.13 a	3.41 ± 0.11 a	0.274 ± 0.01 a	22.89 ± 0.19 a	4.54 ± 0.08
	T5 + 15 mM Ca	T8	7.05 ± 0.27 c	4.46 ± 0.07 d	2.80 ± 0.20 b	0.233 ± 0.01 c	21.86 ± 0.22 c	4.40 ± 0.55
Binatomato-5	Control	T1	4.90 ± 0.51 i	3.21 ± 0.12 k	2.45 ± 0.19 def	0.165 ± 0.00 j	17.90 ± 0.19 i	4.46 ± 0.08
	5 mM Ca	T2	4.94 ± 0.16 i	3.25 ± 0.08 k	2.47 ± 0.11 def	0.169 ± 0.00 j	19.03 ± 0.11 g	4.48 ± 0.23
	10 mM Ca	T3	5.01 ± 0.26 i	3.32 ± 0.6 j	2.51 ± 0.19 cdef	0.173 ± 0.00 ij	19.23 ± 0.23 g	4.54 ± 0.21
	15 mM Ca	T4	4.90 ± 0.15 i	3.07 ± 0.12 l	2.38 ± 0.18 ef	0.166 ± 0.00 j	18.08 ± 0.09 h	4.53 ± 0.22
	12 dS m^−1^ NaCl	T5	5.95 ± 0.33 ef	3.58 ± 0.11 i	2.65 ± 0.09 bcde	0.206 ± 0.00 e	21.05 ± 0.13 d	4.22 ± 0.09
	T5 + 5 mM Ca	T6	6.17 ± 0.25 de	3.67 ± 0.13 h	2.71 ± 0.11 bcd	0.211 ± 0.00 de	21.44 ± 0.19 cd	4.30 ± 0.17
	T5 + 10 mM Ca	T7	6.36 ± 0.33 d	3.88 ± 0.15 f	2.83 ± 0.17 b	0.218 ± 0.01 d	21.71 ± 0.22 c	4.47 ± 0.11
	T5 + 15 mM Ca	T8	6.01 ± 0.33 e	3.32 ± 0.008 j	2.27 ± 0.09 f	0.187 ± 0.00 fgh	20.01 ± 0.13 fg	4.31 ± 0.08
CV (%)			2.34	2.80	6.12	2.68	1.64	5.32
LSD_(0.05)_			0.234	0.051	0.281	0.009	0.545	0.394

Mean values followed by different letters in the same column are significantly different from each other at 5% level of significance. Means of five replicates are shown. CV: coefficient of variation; LSD: least significant difference at 5% level; SD: standard deviation.

## Data Availability

The data are contained within the article.
